# Gastrointestinal adverse events associated with semaglutide: A pharmacovigilance study based on FDA adverse event reporting system

**DOI:** 10.3389/fpubh.2022.996179

**Published:** 2022-10-20

**Authors:** Yamin Shu, Xucheng He, Pan Wu, Yanxin Liu, Yufeng Ding, Qilin Zhang

**Affiliations:** ^1^Department of Pharmacy, Tongji Hospital, Tongji Medical College, Huazhong University of Science and Technology, Wuhan, China; ^2^Department of Pharmacy, Pengzhou Second People's Hospital, Pengzhou, China; ^3^Department of Pharmacy, Chengfei Hospital, Chengdu, China; ^4^Department of Pharmacy, Pengzhou People's Hospital, Pengzhou, China; ^5^Department of Pharmacy, Union Hospital, Tongji Medical College, Huazhong University of Science and Technology, Wuhan, China

**Keywords:** data mining, FAERS, gastrointestinal adverse events, pharmacovigilance, semaglutide

## Abstract

**Background:**

Semaglutide was approved for treatment of type 2 diabetes mellitus (T2DM) and chronic weight management in obesity or overweight adults. However, real-world data regarding its long-term gastrointestinal safety and tolerability in large sample population are incomplete. We evaluated semaglutide-associated gastrointestinal safety signals by data mining of the FDA pharmacovigilance database.

**Methods:**

Reporting odds ratio (ROR) was employed to quantify the signals of semaglutide-related gastrointestinal adverse events (AEs) from 2018 to 2022. Serious and non-serious cases were compared by Mann-Whitney *U* test or Chi-squared (χ^2^) test, and signals were prioritized using a rating scale.

**Results:**

We identified 5,442 cases of semaglutide-associated gastrointestinal AEs, with 45 signals detected, ranging from a ROR_025_ of 1.01 (hypoaesthesia oral) to 42.03 (eructation), among which 17 AEs were identified as new and unexpected signals. Patient age (*p* < 0.001) and body weight (*p* = 0.006) rather than sex (*p* = 0.251) might be associated with an increased risk of gastrointestinal AEs severity. Notably, the association between semaglutide and gastrointestinal disorders remained when stratified by age, body weight, sex and reporter type. One strong, 22 moderate and 22 weak clinical priority signals were defined. The median time-to-onset (TTO) for strong clinical priority signal was 23 days, while for moderate and weak, they were 6 and 7 days, respectively. All of the disproportionality signals had early failure type features, suggesting that the risk of gastrointestinal AEs occurrence gradually decreased over time.

**Conclusion:**

Our study provided a deeper and broader understanding of semaglutide's gastrointestinal safety profiles, which would help healthcare professionals to mitigate the risk of gastrointestinal AEs in clinical practice.

## Introduction

Glucagon-like peptide-1 receptor agonists (GLP-1RAs) are a kind of incretin analogs that can control blood glucose by increasing glucose-dependent insulin secretion, improving insulin resistance, slowing gastric empting, inhibiting glucagon release, and reducing appetite (1, 2). It has been widely used in the treatment of type 2 diabetes mellitus (T2DM) due to its significant hypoglycemic effect. Semaglutide, a novel GLP-1RA with an extended half-life of ~1 week, was approved by the US Food and Drug Administration (FDA) in December 2017 for the treatment of T2DM in adult (3). Subsequently, in January 2020, the FDA expanded the indication of semaglutide to include T2DM adults with cardiovascular disease based primarily on the SUSTAIN-6 clinical trial that showed a statistically significant reduction in the risk of cardiovascular events in the semaglutide group compared with placebo group (6.6 vs. 8.9%, hazard ratio, 0.74; 95% confidence interval [CI], 0.58–0.95; *p* < 0.001) (4, 5). GLP-1RAs was recommended to be added to the treatment of patients with T2DM who are at high risk for adverse cardiovascular events by the European Association for the Study of Diabetes (EASD) and the American Diabetes Association (ADA) in the 2019 guidelines (6). Recently, in June 2021, the FDA approved semaglutide again for long-term weight management in obesity or overweight adults, which was the second GLP-1RA to be approved for weight loss after liraglutide.

After the approval of semaglutide, the post-marketing concerns have been raised about its long-term safety and tolerability for clinical use. The most common adverse events (AEs) reported with GLP-1RAs were gastrointestinal disorders, such as nausea, diarrhea, vomiting, and abdominal pain, etc (7–9). In addition, GLP-1RAs use may be associated with an increased risk of acute pancreatitis and thyroid cancer, and the FDA has warned the public about these AEs (10–12). A systematic review and network meta-analysis published in 2014 revealed that, all GLP-1RAs dose regimens distinctly increased the incidence of gastrointestinal AEs compared with placebo or conventional treatment (7). Another previous real-world disproportionality analysis of GLP-1RAs-associated gastrointestinal AEs based on the FDA Adverse Event Reporting System (FAERS) from 2018 to 2020 included 2,047 cases of semaglutide and 4,075 cases of liraglutide for the treatment of obesity (13). This study demonstrated that semaglutide had a higher pooled ROR and later pooled time-to-onset median of gastrointestinal AEs compared with those of liraglutide. The serious outcomes of gastrointestinal AEs had not been reported and compared. Two years after this study, with the widespread use of semaglutide, a systematic and comprehensive introduction of semaglutide-induced AEs changes will be helpful to clinicians and pharmacovigilance specialists. Hence, the gastrointestinal AEs associated with semaglutide are the special focus of this study.

In the present study, the disproportionality analysis was used to analyze the safety data of semaglutide to quantitative gastrointestinal positive signals based on FAERS database, which was a global, public and accessable pharmacovigilance database (14, 15). Further, stratification analysis, clinical priority of signals, time-to-onset, and the serious outcomes were performed to detect the characteristics of semaglutide-associated gastrointestinal AEs.

## Methods

### Study design and data sources

The study is designed as an observational, retrospective disproportionality analysis, a validated concept in pharmacovigilance to assess whether an association is likely to exist between semaglutide and gastrointestinal AEs. It measures the occurrence of target AEs associated with a drug compared to all other drugs in the FAERS database (16). The FAERS data were downloaded from the FAERS Quarterly Data Extract Files, available at https://fis.fda.gov/extensions/FPD-QDE-FAERS/FPD-QDE-FAERS.html. According to the FDA approval time of semaglutide, all reports recorded in FAERS covering the period from the first quarter (Q1) of 2018–2022 Q1, were included in our analysis.

### Data extraction and descriptive analysis

The FAERS database consisted of seven data files: patient demographic information (DEMO), drug information (DRUG), adverse event information (REAC), patient outcome information (OUTC), report source information (RPSR), drug therapy date information (THER), and drug indication (INDI) (17). In addition, the cases deleted by FDA or manufacturers for various reasons including combining cases, were shown in Deleted files. All data were imported into MySQL software (v8.0; Oracle, Sweden), and the deduplication process was performed before statistical analysis. The primary ID was the primary link field (primary key) between different data files, and the case ID was chosen as the key filter in our study to remove duplicate records (18). We identified cases using generic name (semaglutide in drugname and prod_ai columns) and trade name (OZEMPIC, RYBELSUS, and WEGOVY in drugname column) in the DRUG file, and chose the role_cod as PS (Primary suspected). Moreover, we performed the analysis of concomitant medication. Combination therapy is defined as concurrent administration of semaglutide and other drugs for T2DM or obesity, which implies that in the same report if semaglutide is the PS, the other drugs are the “secondary suspect”, “concomitant” or “interacting”. We further checked the reports manually to select the highest primary ID, when the case ID was the same. AEs in FAERS are coded using the preferred term (PT) from standardized Medical Dictionary for Regulatory Activities (MedDRA), which contains 27 system organ classes (SOCs). Further, a PT can be linked to more than one SOC in MedDRA. Accordingly, MedDRA 24.0 was used to classify AEs in each report to the corresponding SOC levels in MySQL 8.0. All the PTs below the SOC of gastrointestinal disorders (SOC: 10017947) in the FAERS database were included in our study.

**Figure 1:**
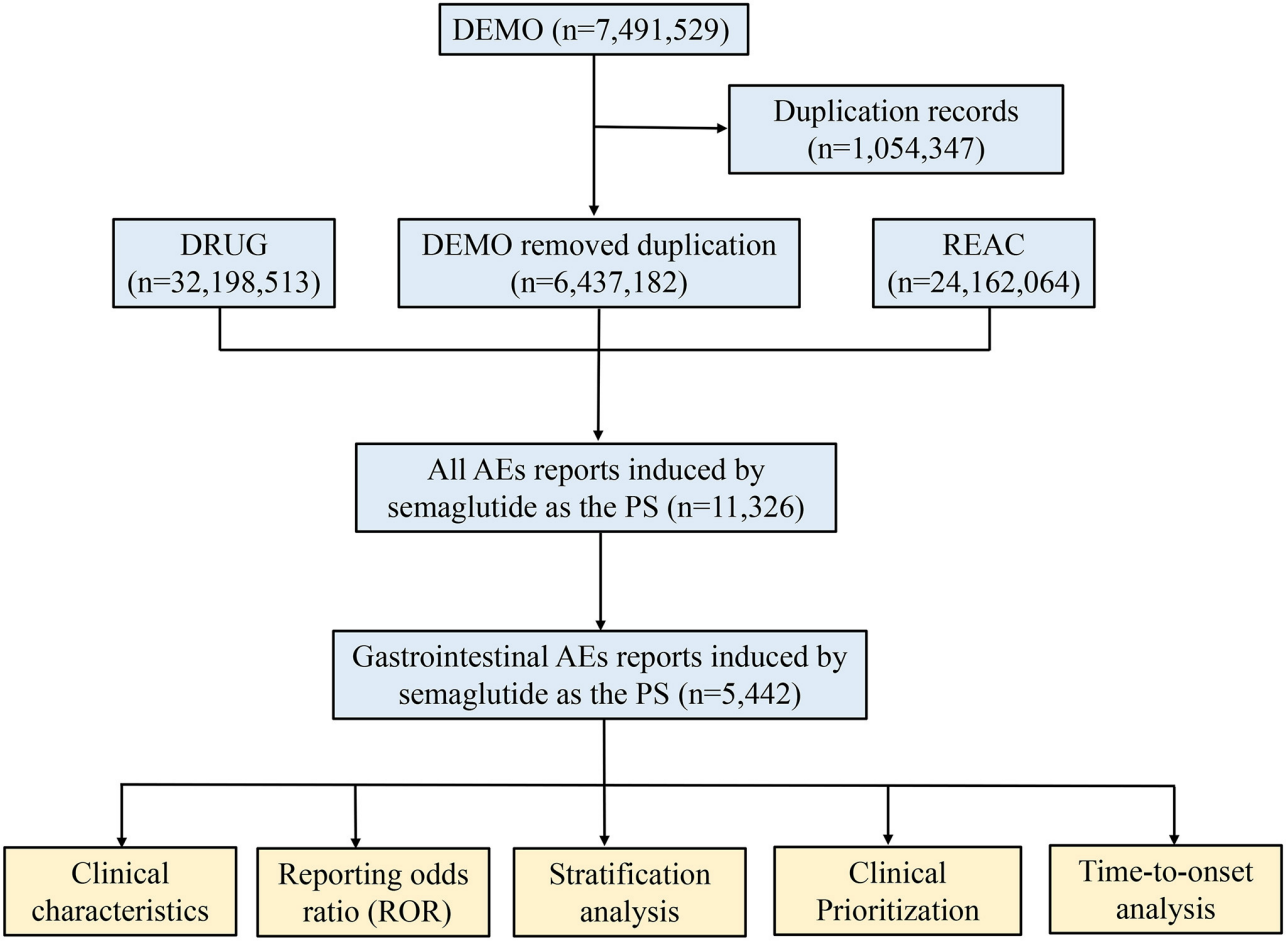
The process of selecting semaglutide-associated gastrointestinal adverse events from food and drug administration adverse event reporting database.

Subsequently, the clinical characteristics of reports were described in detail, if the data were available, including gender, age, weight, reporting area, indications, outcomes and reporters, etc. It is worth noting that the serious outcomes include death, life-threatening, hospitalization, disability, and other serious outcomes. Nevertheless, total serious outcomes may exceed the total number of reports because some cases list more than one serious outcome. A flow diagram including the multi-step process of data extraction, processing, and analysis was shown in Figure 1.

### Statistical analysis

The reporting odds ratio (ROR), one of the algorithms used in disproportionality analysis was based on the 2 × 2 table calculation principle (Supplementary Table S1). All gastrointestinal AEs with at least ten reports were selected to reduce the likelihood of false positives. We then performed signal strength of reports of semaglutide at both PT (gastrointestinal AEs) and SOC levels in FAERS database, and a positive signal was considered when the lower limit of the ROR 95% confidence interval (CI) exceeded one (19). The serious and non-serious reports were also compared to clarify the severity of the detected safety signals and identify risk factors (gender, age and weight) in patients. Proportions were compared using a Pearson's chi-squared (χ^2^) or Fisher's exact test, and Mann-Whitney *U* test was applied for continuous non-normal distribution data, such as age and weight. Data were analyzed using SPSS (v22.0; IBM Corp., Armonk, NY, United States), and statistical significance was set at *p* < 0.05. To further explore the influence of different stratification regimens on the correlation between semaglutide and gastrointestinal disorders, we performed stratification analysis by gender (female and male), age (18 ≤ and ≤ 64, >65 years), weight (<80, 80 ≤ and ≤ 100, >100 kg) and reporters (healthcare professional and consumer) separately.

### Clinical prioritization of signals

A semiquantitative score was employed to prioritize disproportionality signals in five features, including number of reports, the lower limit of the ROR 95% CI (ROR_025_) values, proportion of death outcomes, assessment as designated medical events (DMEs) or important medical events (IMEs), and evidence evaluation (19, 20). According to three levels of clinical importance, a composite score between 0–4, 5–7, and 8–10 was identified as AEs with weak, moderate or strong clinical priority, respectively. The detailed information was shown in Supplementary Table S2.

### Time-to-onset analysis

Time-to-onset (TTO) was defined as the interval between the AEs onset date (EVENT_DT in DEMO file) and start date of semaglutide use (START_DT in THER file) (17, 18). In order to ensure the accuracy of this calculation, reports with input errors (EVENT_DT earlier than START_DT), inaccurate date entries and missing specific data were excluded. The medians, quartiles and the Weibull shape parameter (WSP) test were used to evaluate the TTO in our study (21, 22). The TTO statistical analysis was conducted using the WSP test, which could determine the varying ratio of incidence of AEs. The shape of the Weibull distribution was described by two parameters: scale (α) and shape (β). In order to predict the risk of increase or decrease of these AEs over time, we calculated the median TTO and WSP of signals with strong, moderate or weak clinical priority after semaglutide use. The selection of parameters and criteria for evaluation were described in previous studies (21, 22). All WSP tests were performed by Minitab statistical software (v20.0; Minitab LLC, State College, PA, United States).

## Results

### Descriptive analysis

During the study period, a total of 6,437,182 AE reports were obtained from the FAERS database after exclusion of duplicates, containing 11,326 semaglutide-related gastrointestinal AEs in 5,442 patients. The detailed clinical characteristics were summarized in Table 1. Gender data were available for 5,312 patients, and females accounted for a larger proportion than males (3,064 vs. 2,248). The gastrointestinal AEs treated with semaglutide were more likely to occur in middle-aged patients (18–65 years, *n* = 1,962, 56.73%) than the elderly patients (>65 years, *n* = 1,494, 43.21%). 1,005 patients reported weight data, with the median weight of 96.16 kg. The report proportions of body weight >100, 80–100, and <80 kg were 42.39, 34.43, and 23.18%, respectively. Serious outcomes of gastrointestinal and overall AEs reports were recorded in 1,778 and 3,601 cases, including 40 (2.25%) and 102 (2.83%) deaths, respectively. Other serious events and hospitalizations were the most frequently reported serious outcomes of semaglutide treatment, occurring in 1,103 (62.04%), 772 (43.42%) gastrointestinal AEs and 2,311 (64.18%), 1,430 (39.71%) overall AEs, respectively. Additionally, 40.25% of the gastrointestinal reports were submitted by healthcare professionals (*n* = 2,183), compared to 59.75% reported by consumers (*n* = 3,240). T2DM was the most reported indication (*n* = 1,656, 59.83%), followed by other unspecified diabetes mellitus (*n* = 715, 25.83%) and obesity (*n* = 133, 4.80%). The country with the most gastrointestinal AEs reports was USA (*n* = 4,864, 89.38%). The Supplementary Table S3 showed metformin hydrochloride and insulin glargine were the top 2 combination drugs for semaglutide-associated gastrointestinal AEs, with 564 (10.36%) and 170 (3.12%) cases, respectively.

**Table 1:** Clinical characteristics of patients with semaglutide-associated gastrointestinal adverse events.

**Characteristics**	**Semaglutide induced gastrointestinal AEs**		**Semaglutide induced overall AEs**	
	**(*****n*** = **5,442)**		**(*****n*** = **11,326)**	
	**Available number**	**Value**	**Available number**	**Value**
Gender, *n* (%)	5,312 (97.61%)	–	11,053	–
Female	–	3,064 (57.68%)	–	6,519 (58.98%)
Male	–	2,248 (42.32%)	–	4,534 (41.02%)
Age (years), *n* (%)	3,458 (63.54%)	–	6531 (57.66%)	–
<18	–	2 (0.06%)	–	6 (0.09%)
18 ≤ and ≤ 65	–	1,962 (56.73%)	–	3,771 (57.74%)
>65	–	1,494 (43.21%)	–	2,754 (42.17%)
Median (years)	–	63	–	63
Weight (kg), *n* (%)	1,005 (18.47%)	–	1,676 (14.80%)	–
<80	–	233 (23.18%)	–	396 (23.63%)
80 ≤ and ≤ 100	–	346 (34.43%)	–	594 (35.44%)
>100	–	426 (42.39%)	–	686 (40.93%)
Median (kg)	–	96.16	–	95.36
Reported countries, *n* (%)	5,442 (100%)	–	11,326 (100%)	–
US	–	4,864 (89.38%)	–	10,032 (88.57%)
Non-US	–	578 (10.62%)	–	1,294 (11.43%)
Indications, *n* (%)	2,768 (50.86%)	–	5,122 (45.22%)	–
Type 2 diabetes mellitus	–	1,656 (59.83%)	–	3,158 (61.66%)
Diabetes mellitus	–	715 (25.83%)	–	1,154 (22.53%)
Obesity	–	133 (4.80%)	–	308 (6.01%)
Others	–	264 (9.54%)	–	502 (9.80%)
Outcomes, *n* (%)	5,442 (100%)	–	11,326 (100%)	–
Non-serious outcome	–	3,664 (67.33%)	–	7,725 (68.21%)
Serious outcome	–	1,778 (32.67%)	–	3,601 (31.79%)
Death	–	40 (2.25%)	–	102 (2.83%)
Life-threatening	–	53 (2.98%)	–	98 (2.72%)
Hospitalization	–	772 (43.42%)	–	1,430 (39.71%)
Disability	–	57 (3.21%)	–	132 (3.67%)
Other serious outcomes	–	1,103 (62.04%)	–	2,311 (64.18%)
Reporters, *n* (%)	5,423 (99.65%)	–	11,292 (99.70%)	–
Health professional	–	2,183 (40.25%)	–	4,424 (39.18%)
Consumer	–	3,240 (59.75%)	–	6,868 (60.82%)
Reporting year, *n* (%)	5,442 (100%)	–	11,326 (100%)	–
2022 Q1^*^	–	949 (17.44%)	–	2,257 (19.93%)
2021	–	1,847 (33.94%)	–	3,819 (33.72%)
2020	–	1,501 (27.58%)	–	2,901 (25.61%)
2019	–	691 (12.70%)	–	1,305 (11.52%)
2018	–	454 (8.34%)	–	1,044 (9.22%)

* The first quarter of 2022.

AEs, adverse events; n, number of cases.

### Disproportionality analysis

**Figure 2:**
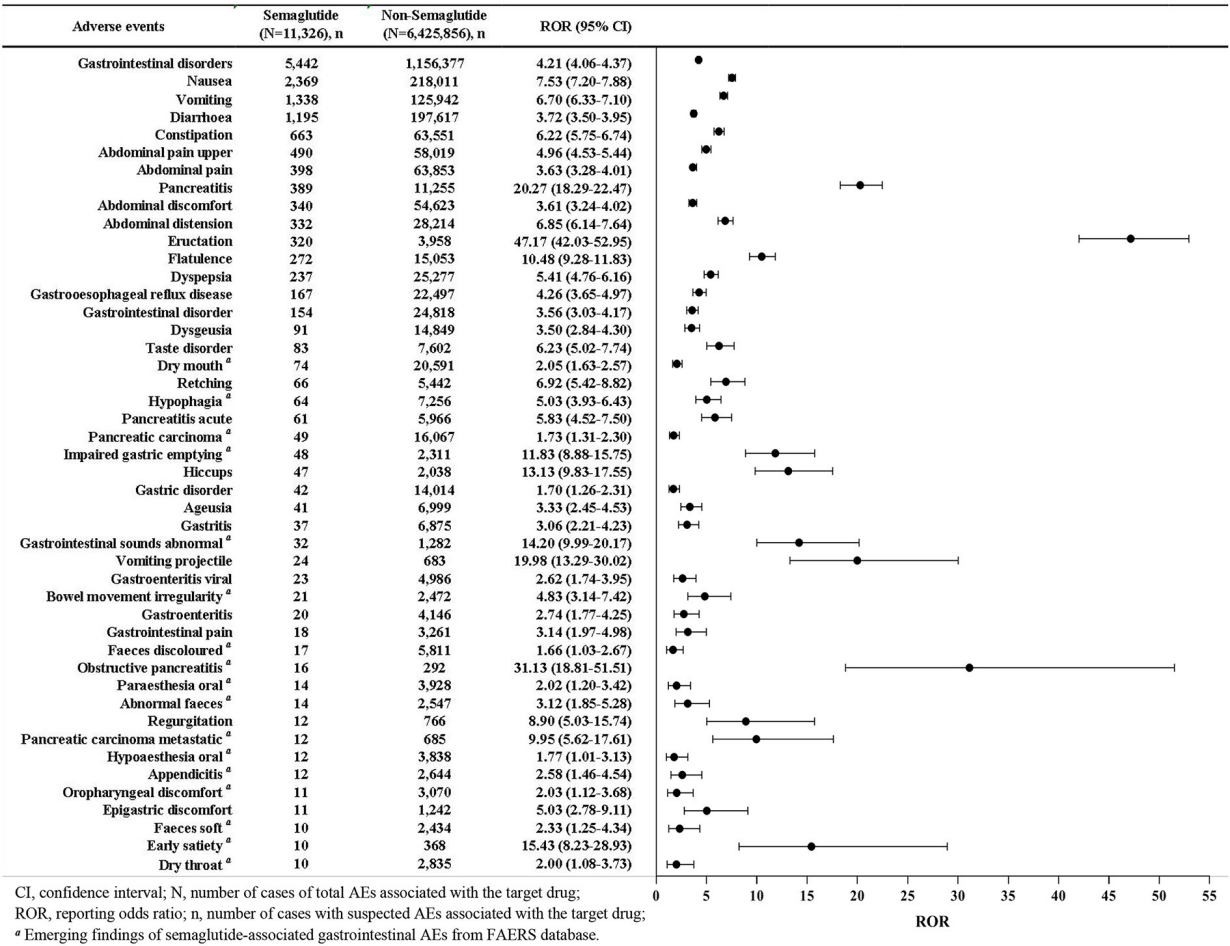
Reporting odds ratios (ROR) with 95% CI for all positive semaglutide-related gastrointestinal AEs.

A total of 45 different PTs of the gastrointestinal AEs associated with semaglutide were reported in FAERS database in at least 10 cases (Figure 2). The most frequently reported gastrointestinal AEs were nausea (*n* = 2,369), vomiting (*n* = 1,338), diarrhea (*n* = 1,195), constipation (*n* = 663), abdominal pain upper (*n* = 490), abdominal pain (*n* = 398), and pancreatitis (*n* = 389). Results of the disproportionality analysis of the gastrointestinal AEs associated with semaglutide were presented in Figure 2. The report frequency of gastrointestinal disorders related to semaglutide was significantly higher than that of non-semaglutide in the overall database with the ROR of 4.21. The 45 semaglutide-related gastrointestinal AEs also showed statistically significant signal strengths as compared to non-semaglutide-associated gastrointestinal AEs, with values of signals ranging from a ROR_025_ of 1.01 (hypoaesthesia oral) to 42.03 (eructation). Among 27 SOCs, results of Supplementary Table S4 demonstrated that SOC of gastrointestinal disorders treated with semaglutide exhibited the strongest association owing to its highest ROR with 4.21 (4.06–4.37).

### Serious vs. non-serious cases

As shown in Table 2, there were statistically significant differences in age (61 vs. 64.5 years; *p* < 0.001) and body weight (95 vs. 98.1 kg; *p* = 0.006) between severe and non-severe cases of gastrointestinal AEs patients receiving semaglutide. However, men and women proportions did not differ between the two groups (χ^2^ = 1.316, *p* = 0.251). 27 AEs were more likely to be reported as serious AEs with *p* < 0.05, such as nausea (χ^2^ = 178.202, *p* < 0.001), vomiting (χ^2^ = 4.862, *p* = 0.027), pancreatitis (χ^2^ = 533.618, *p* < 0.001) and constipation (χ^2^ = 16.966, *p* < 0.001), etc. Another 18 AEs were more tended to be reported as non-serious AEs with *p* > 0.05, such as diarrhea (χ^2^ = 3.405, *p* = 0.065), gastrooesophageal reflux disease (χ^2^ = 1.606, *p* = 0.205), dry mouth (χ^2^ = 3.208, *p* = 0.073), retching (χ^2^ = 3.004, *p* = 0.083), etc. Of note, all AEs outcomes for pancreatic carcinoma metastatic (*n* = 12) were severe, while all oropharyngeal discomfort (*n* = 11) were non-severe.

**Table 2:** Differences in clinical characteristics of serious and non-serious reports.

	**Serious cases**	**Non-serious cases**	**Statistic**	***p*** **-value**
Gender, *n* (%)	–	–	–	–
Female	990 (32.31)	2,074 (67.69)	1.316^b^	0.251^a^
Male	760 (33.81)	1,488 (66.19)		
Age, years (median, IQR)	61 (52–70)	64.5 (56–72)	−6.723^d^	< 0.001^c^
Weight, kg (median, IQR)	95 (80.00–110.72)	98.1 (81.77–117.93)	−2.743^d^	0.006^c^
Types of AEs, *n* (%)	–	–	–	–
Nausea	545 (30.65)	1,824 (49.78)	178.202^b^	< 0.001^a^
Vomiting	470 (26.43)	868 (23.69)	4.862^b^	0.027^a^
Diarrhea	364 (20.47)	831 (22.68)	3.405^b^	0.065^a^
Pancreatitis	333 (18.73)	56 (1.53)	533.618^b^	< 0.001^a^
Constipation	170 (9.56)	493 (13.46)	16.966^b^	< 0.001^a^
Abdominal pain	163 (9.17)	235 (6.41)	13.393^b^	< 0.001^a^
Abdominal pain upper	140 (7.87)	350 (9.55)	4.116^b^	0.042^a^
Eructation	75 (4.22)	245 (6.69)	13.180^b^	< 0.001^a^
Flatulence	67 (3.77)	205 (5.59)	8.412^b^	0.004^a^
Abdominal distension	65 (3.66)	267 (7.29)	27.556^b^	< 0.001^a^
Abdominal discomfort	57 (3.21)	283 (7.72)	41.717^b^	< 0.001^a^
Pancreatitis acute	54 (3.04)	7 (0.19)	87.487^b^	< 0.001^a^
Dyspepsia	49 (2.76)	188 (5.13)	16.212^b^	< 0.001^a^
Gastrooesophageal reflux disease	47 (2.64)	120 (3.28)	1.606^b^	0.205^a^
Pancreatic carcinoma^f^	46 (2.59)	3 (0.08)	84.205^b^	< 0.001^a^
Gastrointestinal disorder	33 (1.86)	121 (3.30)	9.108^b^	0.003^a^
Hypophagia^f^	29 (1.63)	35 (0.96)	4.704^b^	0.03^a^
Impaired gastric emptying^f^	26 (1.46)	22 (0.60)	10.172^b^	0.001^a^
Gastritis	25 (1.41)	12 (0.33)	20.622^b^	< 0.001^a^
Gastroenteritis	18 (1.01)	2 (0.05)	29.991^b^	< 0.001^a^
Taste disorder	17 (0.96)	66 (1.80)	5.694^b^	0.017^a^
Dry mouth^f^	17 (0.96)	57 (1.56)	3.208^b^	0.073^a^
Retching	15 (0.84)	51 (1.39)	3.004^b^	0.083^a^
Obstructive pancreatitis^f^	15 (0.84)	1 (0.03)	27.215^b^	< 0.001^a^
Dysgeusia	15 (0.84)	76 (2.07)	11.025^b^	0.001^a^
Pancreatic carcinoma metastatic^f^	12 (0.67)	0 (0.00)	–	< 0.001^e^
Appendicitis^f^	10 (0.56)	2 (0.05)	–	< 0.001^e^
Hiccups	9 (0.51)	38 (1.04)	3.941^b^	0.047^a^
Gastric disorder	9 (0.51)	33 (0.90)	2.432^b^	0.119^a^
Ageusia	9 (0.51)	32 (0.87)	2.158^b^	0.142^a^
Vomiting projectile	8 (0.45)	16 (0.44)	0.005^b^	0.945^a^
Gastroenteritis viral	7 (0.39)	16 (0.44)	0.053^b^	0.819^a^
Gastrointestinal pain	6 (0.34)	12 (0.33)	0.004^b^	0.952^a^
Feces discolored^f^	6 (0.34)	11 (0.30)	0.053^b^	0.817^a^
Bowel movement irregularity^f^	5 (0.28)	16 (0.44)	0.753^b^	0.386^a^
Epigastric discomfort	4 (0.22)	7 (0.19)	–	0.757^e^
Gastrointestinal sounds abnormal^f^	3 (0.17)	29 (0.79)	7.942^b^	0.005^a^
Dry throat^f^	3 (0.17)	7 (0.19)	–	1.000^e^
Abnormal feces^f^	3 (0.17)	11 (0.30)	–	0.569^e^
Feces soft^f^	2 (0.11)	8 (0.22)	–	0.514^e^
Early satiety^f^	2 (0.11)	8 (0.22)	–	0.514^e^
Regurgitation	1 (0.06)	11 (0.30)	–	0.119^e^
Paraesthesia oral^f^	1 (0.06)	13 (0.35)	–	0.046^e^
Hypoaesthesia oral^f^	1 (0.06)	11 (0.30)	–	0.119^e^
Oropharyngeal discomfort^f^	0 (0.00)	11 (0.30)	–	0.020^e^

The AEs listed above were AEs with significant signal strengths.

a Proportions were compared using Pearson χ^2^ test.

b The χ^2^ statistic of the Pearson chi-square test.

c Mann-Whitney U test.

d The Z statistic of the Mann-Whitney U test.

e Fisher's exact test.

f Emerging findings of semaglutide-associated gastrointestinal AEs from FAERS database.

p-value < 0.05 were considered statistically significant.

### Stratification analysis

**Figure 3:**
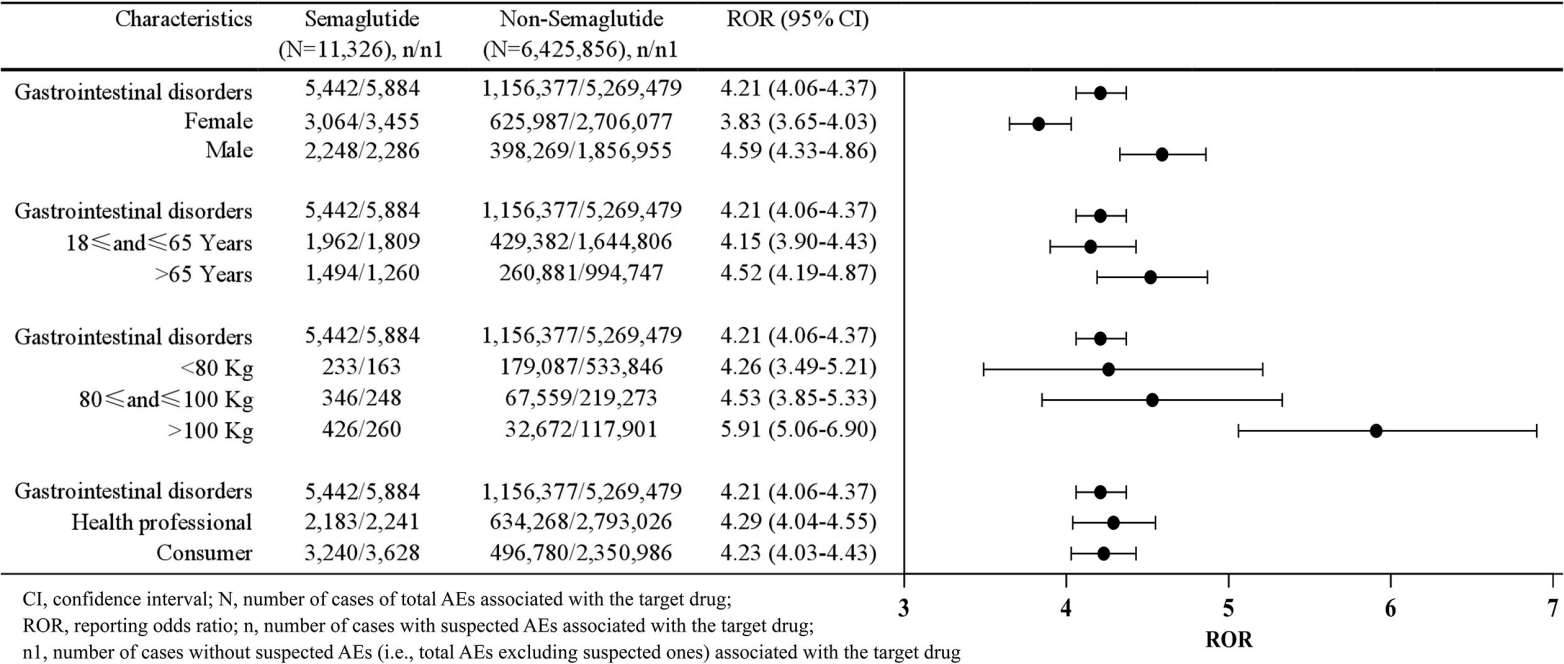
Stratification analysis of semaglutide-induced gastrointestinal disorders.

Four different stratification strategies were employed to increase the robustness of the results. As shown in Figure 3, after SOC of gastrointestinal disorders was assessed separately by sex, age, body weight and reporters' type, the lower limits of ROR values were >1 in all stratified subgroups, revealing that there was still a strong statistical correlation between semaglutide and gastrointestinal disorders. Since there were only 2 patients younger than 18 years old, they did not show significant signal strength with ROR_025_ 0.54. Thus, these populations were excluded from the stratification analysis.

### Clinical prioritization of the disproportionality signals

Overall, 11 out of the 45 AEs (24.44%) showing statistically significant disproportionality were categorized as IMEs, and only 2 (4.44%) as DMEs, including pancreatitis and pancreatitis acute (Table 3). Based on the clinical priority assessment results, 1 (2.22%), 22 (48.89%), and 22 (48.89%) AEs were identified as strong, moderate and weak clinical priority, respectively. Pancreatitis (*n* = 389, ROR_025_ = 18.29) was classified as strong clinical priority with the highest priority score 8. As for the evaluation of relevant evidence, 22 AEs presented a strong clinical evidence with “++”. Of note, 17 new and unexpected AEs which showed statistically significant RORs and were not presented in the drug label were also detected in our data analysis. The new AE signals were marked in Figure 2.

**Table 3:** Clinical priority assessing results of disproportionality signals.

**PTs**	***n***	**ROR_025_**	**Death (*n*)**	**IMEs/DMEs**	**Relevant evidence** **evaluation**	**Priority level** **(score)**
Nausea	2,369	7.20	0	NA	++	Moderate (6)
Vomiting	1,338	6.33	3	NA	++	Moderate (6)
Diarrhea	1,195	3.50	0	NA	++	Moderate (5)
Constipation	663	5.75	0	NA	++	Moderate (6)
Abdominal pain upper	490	4.53	2	NA	++	Moderate (5)
Abdominal pain	398	3.28	1	NA	++	Moderate (5)
Pancreatitis	389	18.29	2	DME	++	Strong (8)
Abdominal discomfort	340	3.24	0	NA	++	Moderate (5)
Abdominal distension	332	6.14	1	NA	++	Moderate (6)
Eructation	320	42.03	0	NA	++	Moderate (6)
Flatulence	272	9.28	0	NA	++	Moderate (6)
Dyspepsia	237	4.76	0	NA	++	Moderate (5)
Gastrooesophageal reflux disease	167	3.65	0	NA	++	Moderate (5)
Gastrointestinal disorder	154	3.03	1	NA	++	Moderate (5)
Dysgeusia	91	2.84	0	IME	++	Moderate (6)
Taste disorder	83	5.02	0	IME	++	Moderate (7)
Dry mouth^a^	74	1.63	0	NA	–	Weak (2)
Retching	66	5.42	0	NA	++	Moderate (6)
Hypophagia^a^	64	3.93	0	NA	–	Weak (3)
Pancreatitis acute	61	4.52	1	DME	++	Moderate (7)
Pancreatic carcinoma^a^	49	1.31	7	NA	++	Weak (3)
Impaired gastric emptying^a^	48	8.88	0	IME	+	Moderate (5)
Hiccups	47	9.83	0	NA	++	Moderate (5)
Gastric disorder	42	1.26	0	NA	++	Weak (3)
Ageusia	41	2.45	0	IME	+	Weak (4)
Gastritis	37	2.21	0	IME	++	Moderate (5)
Gastrointestinal sounds abnormal^a^	32	9.99	0	NA	–	Weak (3)
Vomiting projectile	24	13.29	0	NA	+	Weak (4)
Gastroenteritis viral	23	1.74	0	IME	+	Weak (3)
Bowel movement irregularity^a^	21	3.14	0	NA	–	Weak (2)
Gastroenteritis	20	1.77	0	IME	+	Weak (3)
Gastrointestinal pain	18	1.97	0	NA	+	Weak (2)
Feces discolored^a^	17	1.03	0	NA	–	Weak (1)
Obstructive pancreatitis^a^	16	18.81	0	IME	+	Moderate (5)
Paraesthesia oral^a^	14	1.20	0	IME	–	Weak (2)
Abnormal feces^a^	14	1.85	0	NA	–	Weak (1)
Pancreatic carcinoma metastatic^a^	12	5.62	3	IME	–	Moderate (5)
Regurgitation	12	5.03	0	NA	+	Weak (4)
Hypoaesthesia oral^a^	12	1.01	0	IME	–	Weak (2)
Appendicitis^a^	12	1.46	0	NA	–	Weak (1)
Epigastric discomfort	11	2.78	0	NA	+	Weak (3)
Oropharyngeal discomfort^a^	11	1.12	0	NA	–	Weak (1)
Early satiety^a^	10	8.23	0	NA	–	Weak (3)
Feces soft^a^	10	1.25	0	NA	–	Weak (1)
Dry throat^a^	10	1.08	0	NA	–	Weak (1)

A priority score between 8 and 10, 5–7 or 0–4 represents the signal with strong, moderate or weak clinical priority, respectively.

NA, Not Applicable (for relevant criterias); n, number of cases; PTs, Preferred Terms; ROR_025_, the lower limit of 95% confidence interval of ROR.

a Emerging finding of semaglutide-associated gastrointestinal AEs from FAERS database.

### Time-to-onset analysis

Results of time-to-onset and WSP analysis for the strong, moderate and weak clinical priorities signals were shown in Table 4. The median onset time of strong, moderate and weak signals related to semaglutide was 23 (IQR 2–92), 6 (IQR 0–31), and 7 (IQR 0–32.25) days, respectively. Notably, the moderate and weak clinical priority signals tended to occur earlier than those strong AEs signals. In the assessment of WSP analysis, all shape parameters β and their 95% CI upper limits were <1, suggesting that these strong, moderate and weak clinical priority signals had early failure types.

**Table 4:** Time-to-onset analysis for signals with strong/moderate/weak prioritization.

**Prioritization**				**Weibull distribution**				**Failure type**
	**Cases**	**TTO (days)**		**Scale parameter**		**Shape parameter**		
	***n***	**Median (IQR)**	**Min–max**	**α**	**95% CI**	**β**	**95% CI**	
Strong	125	23 (2–92)	0–833	33.45	21.07–53.11	0.40	0.34–0.46	Early failure
Moderate	2,138	6 (0–31)	0–717	8.07	7.35–8.85	0.34	0.33–0.35	Early failure
Weak	229	7 (0–32.25)	0–688	10.11	6.79–15.05	0.33	0.30–0.37	Early failure

n, number of cases with available time-to-onset; IQR, interquartile range; TTO, Time-to-onset. When TTO is 0 days, the adverse event occurred within the same day with the therapy.

## Discussion

This study provided the latest findings of semaglutide-related gastrointestinal safety profiles by post-marketing based on real-world population from FAERS database. Although our results were consistent with previous clinical trials and literature reviews that semaglutide might increase the risk of gastrointestinal disorders, our report presented a more accurate and detailed description and characterization of gastrointestinal AEs spectrum of semaglutide to date, which innovatively added stratification analysis, clinical priority of signals, and the serious outcomes.

### Comparison of safety signals between different studies

Considering the increased number of approved indications and widespread use of semaglutide, gastrointestinal AEs reported a remarkable increase from 2018 to 2021, with the annual reports in 2021 (*n* = 1,847) almost 4 times of 2018 (*n* = 454). A total of 5,442 reports of semaglutide-related gastrointestinal AEs were retained in our study, while Zhou et al. (13) reported only 2,047 cases, and the number of cases reported in each year was unknown. Based on our data prediction, reports of semaglutide will continue to increase in 2022, as there are 949 cases in the first quarter of 2022 alone, which is far above the average quarterly number for 2018–2021. The current study showed the most frequently reported gastrointestinal events were nausea (*n* = 2,369), vomiting (*n* = 1,338), diarrhea (*n* = 1,195) and constipation (*n* = 663), which were corresponding to clinical trials (4, 23). A network meta-analysis about the safety of once-weekly semaglutide in adults demonstrated that semaglutide was associated with a higher risk of gastrointestinal AEs (e.g., nausea, vomiting, diarrhea, and constipation) than placebo (24). The mechanism of semaglutide-associated nausea/vomiting and diarrhea is not fully understood. These gastrointestinal symptoms are thought to be related to GLP-1RA activating central and peripheral GLP-1 receptors and delaying gastric emptying (25). In a previous study, only 13 semaglutide-associated gastrointestinal AEs were reported in 2018–2020 from the FAERS database (13), while 45 significant AEs with at least ten reports were detected in our study, among which 17 AEs were identified as new and unexpected signals. The full list of all new semaglutide-associated gastrointestinal AEs was shown in Figure 2.

### Serious vs. non-serious reports

A pooled analysis of the STEP 1–3 clinical trials of evaluating the gastrointestinal AE profile of semaglutide revealed that most gastrointestinal AEs reported were non-serious (99.5% of AEs) and mild-to-moderate (98.1%) in severity, which did not necessitate dose reduction or discontinuation (26). Nevertheless, in the current study, there were significant differences (*p* < 0.05) in 27 AEs among the 45 gastrointestinal AEs, when compared between serious cases and non-serious cases. Patient age (*p* < 0.001) and body weight (*p* = 0.006) rather than sex (*p* = 0.251) might be associated with an increased risk of gastrointestinal AEs severity. Semaglutide-associated gastrointestinal AEs seemed to predominately affect females (57.68%) (Table 1), which was in line with the finding that gastrointestinal risks in T2DM occurred more often in females (52.32%) (13). Further comparison of serious and non-serious cases showed that the proportion of serious AEs was similar in males and females (33.81 vs. 32.31%), and there was no statistical difference between the two groups (*p* = 0.251). The association was stronger in males than in females with a higher ROR_025_ value (4.33 vs. 3.65). Few studies have assessed the effect of gender on semaglutide-associated side effects, nor have they explored the exact mechanisms by which gender affects semaglutide-induced gastrointestinal toxicity. Further prospective studies are necessary to determine whether gender is a key factor in clinical practice.

The descriptive analysis in Table 1 indicated that patients aged 18–65 years reported more frequently gastrointestinal AEs (*n* = 1,962, 56.73%) than those aged >65 (*n* = 1,494, 43.21%) and <18 years (*n* = 2, 0.06%), which might be due to the high incidence of diabetes in patients aged 18–65 years (27). Besides, severe cases were reported at significantly younger ages than non-severe cases (median age 61 vs. 64.5 years, *p* < 0.001). These results are slightly different from the findings of previous studies on semaglutide (4, 28). The SUSTAIN 6 clinical trial found that older patients with comorbid conditions were treated with semaglutide for 104 weeks, the incidence of gastrointestinal disorders was somewhat higher (4). An exploratory analysis of evaluating the effect of patient age on the safety of oral semaglutide showed that there was a tendency for higher rates of premature trial product discontinuation due to AEs with increasing age (28). Among patients receiving semaglutide, 86% of those aged ≥65 years reported AEs compared with 76% of those aged 45–65 years and 80% of those aged <45 years (29). Coincidentally, consistent with these studies, our further stratified analysis showed a stronger association with gastrointestinal AEs in subgroups >65 years than in subgroups 18–65 years with a higher ROR_025_ of 4.19. Therefore, slower dose escalations could be used in older patients with comorbidities to help mitigate any AEs that might lead to treatment interruptions. In addition, treatment strategies should be individualized optimally to the patient as recommended in the guidelines.

T2DM is usually associated with obesity and the treatment is based on lifestyle changes to promote weight loss and increase exercise (30). However, it is difficult to adhere to. The vast majority of obese patients tend to choose pharmacotherapy to lose weight. Based on our knowledge and positive results from multiple clinical trials, the FDA approved semaglutide in June 2021 for long-term weight management in overweight or people with obesity (31–33). Recent studies have shown that semaglutide was significantly more effective in weight loss than liraglutide for overweight or obesity without diabetes, and it also exhibited excellent effect in East Asian populations (34, 35). Excitingly, our study detected the indication of semaglutide for obesity with 133 cases. Subsequently, we conducted stratified analysis by body weight (< 80, 80–100 and >100 kg), and found that the subgroup with body weight >100 kg had the highest ROR_025_ value of 5.06, presenting the strongest signal strength. ROR_025_ values of the two subgroups smaller than 80 and 80–100 kg were similar (3.49 and 3.85, respectively), but both showed positive significant disproportionation signals (Figure 3). In addition, patients >100 kg reported more gastrointestinal AEs than those < 80 and 80–100 kg. Hence, the association between semaglutide and gastrointestinal disorders remained when stratified by body weight. However, clinicians may query whether weight losses are the result of these gastrointestinal AEs. Researchers observed that weight loss with semaglutide ranged from 9.6 to 17.1% in participants without gastrointestinal AEs and from 11.4 to 17.7% in those with gastrointestinal AEs, suggesting gastrointestinal AEs appeared to contribute little to the weight-loss benefit of semaglutide, which were consistent with previous studies that the weight-loss effects of semaglutide in patients with obesity and/or T2DM indicated little contribution of gastrointestinal AEs (26, 36, 37).

### Clinical prioritization of the disproportionality signals

In this study, we innovatively used a rating scale to further analyze the disproportionality signals in order to prioritize safety signals and avoid unnecessary warnings. This method might also help clinicians and pharmacovigilance experts improve the accuracy and reliability of positive signals by evaluating current evidence. Our analysis showed the gastrointestinal AEs signal with strong clinical priority was pancreatitis with score 8. In addition, 22 moderate and 22 weak clinical priority signals were defined. Pancreatitis acute (score 7), obstructive pancreatitis (score 5) and pancreatic carcinoma metastatic (score 5) were considered to be moderate clinical priority signals. However, pancreatic carcinoma (score 3) was weak. It was noteworthy that pancreatitis ranked 7th among the top 10 gastrointestinal AEs in terms of the number of reports, showing strong signal strength with ROR 20.27 (18.29–22.47) in the current study (Figure 2). Additionally, significant AEs of pancreatitis acute (*n* = 61, ROR 5.83, 95% CI 4.52–7.50), pancreatic carcinoma (*n* = 49, ROR 1.73, 95% CI 1.31–2.30), obstructive pancreatitis (*n* = 16, ROR 31.13, 95% CI 18.81–51.51), and pancreatic carcinoma metastatic (*n* = 12, ROR 9.95, 95% CI 5.62–17.61) were also observed. Although semaglutide had clearly been reported in clinical trials for increasing the risk of acute pancreatitis and pancreatic cancer, as well as promoting levels of lipase and amylase, several meta-analyses had revealed contrary results (4, 38). Both Nreu et al. (39), Monami et al. (40) found that the incidence of pancreatitis and pancreatic cancer with GLP-1 RAs including semaglutide was not significant from that observed in comparator arms. One systematic review involving 3 RCTs and another involving 9 RCTs similarly revealed semaglutide did not increase the risk of pancreatitis (41, 42). Safety study of injectable semaglutide for type 2 diabetes suggested that the thyroid and pancreatic safety had not been substantiated (43). These conclusions might be biased due to the limited sample size and lack of updated real-world evidence. However, our post-marketing pharmacovigilance analysis based on big data demonstrated a strong association between semaglutide exposure and the occurrence of pancreatitis and pancreatic cancer, further confirming the results of clinical trials. In summary, the safety signal spectrum of semaglutide might change over time as more reports were submitted.

Semaglutide-related pancreatitis was more likely to be reported as a severe AE with strong clinical priority, arousing our great interest to further explore its detailed characteristics. A total of 389, 61, 49, 16, and 12 cases of semaglutide-associated pancreatitis, pancreatitis acute, pancreatic carcinoma, obstructive pancreatitis and pancreatic carcinoma metastatic were extracted from the FAERS database, with 333, 54, 46, 15, and 12 reported as serious cases, respectively (Table 2). Significant differences were observed in these pancreas-associated AEs reports when compared their serious cases with non-serious cases (*p* < 0.001). Furthermore, we detected severe outcomes in 46 cases of pancreatic carcinoma and all cases of pancreatic carcinoma metastatic (*n* = 12), including 7 and 3 deaths, respectively, consistent with a clinical trial that reported 1 pancreatic carcinoma death, which was assessed as being probably related to semaglutide (44). Therefore, due to the potential severity of pancreatitis, clinicians should alert patients to pay attention to the symptoms after administration of semaglutide to mitigate the risk. If pancreatitis is suspected, necessary screening should be performed. If diagnosed, semaglutide should be discontinued immediately and appropriate treatment should be taken.

In addition to pancreatitis, among the moderate clinical priority signals, the most commonly reported semaglutide-related AEs were nausea (score 6), vomiting (score 6), diarrhea (score 5), constipation (score 6), and abdominal pain (score 5), and so on, which were consistent with the drug label. All disproportionality clinical priority signals of semaglutide treatment were listed in Table 3. It was reported that Weibull parameter could be used to predict the period before an AE occured and provided relevant insights for patient pharmacological management in clinical practice (22). In the TTO analysis, the median TTO for pancreatitis was 23 days, while for moderate and weak clinical priority signals, they were 6 and 7 days, respectively. All of the disproportionality signals had early failure type characteristics, suggesting that the majority of patients developed gastrointestinal AEs within 1 week or 1 month of semaglutide treatment, and that the risk of gastrointestinal AEs occurrence gradually decreased over time. This result corresponded to a research showing that the gastrointestinal AEs associated with GLP-1RAs were dose dependent and decline over time (45). The data on time-to-onset for moderate AEs signals were not significantly different from a recent study, indicating the median durations of nausea, diarrhea and vomiting were 8, 3, and 2 days respectively, in the semaglutide 2.4 mg arm (26). A real-world pharmacovigilance study of semaglutide had also demonstrated a similar pooled median time-to-onset of gastrointestinal AEs (7 days, IQR 0–48 days), which further supported the findings of our analysis (13). Hence, careful monitoring in the early period following semaglutide administration might detect most gastrointestinal AEs. Once gastrointestinal AEs are detected in patients, dose adjustment or supportive measures can be adopted to alleviate symptoms and avoid the occurrence of severe AEs.

Among the 45 gastrointestinal AEs, 17 new and unexpected AEs, including dry mouth, hypophagia, gastrointestinal sounds abnormal, bowel movement irregularity, feces discolored, pancreatic carcinoma metastatic, etc, were detected in our pharmacovigilance study, which were not reported in the drug label. The exact effects of semaglutide on these AEs and the mechanisms of this potential association were not completely explored, requiring further clinical investigation. With the widespread presence of semaglutide, gastrointestinal AEs, especially for the newly recorded signals should be flagged as safety alerts by clinicians.

## Limitations

Despite the advantages of real-world data mining strategies utilized in our study based on the FAERS database, there were several limitations inherently by all pharmacovigilance databases. First, the possibility of submitting false, underreported, inaccurate, incomplete, and delayed reports can hardly be solved, which may result in inevitable bias. Second, only cases with adverse events are included in FAERS database. The incidence of gastrointestinal AEs associated with semaglutide cannot be calculated because the total number of populations receiving semaglutide treatment is unknown, i.e., lacking the denominator of drug exposure. Third, the establishment of definite causal relationship between a target drug and AEs is restricted because disproportionality analysis only provides statistical association. Fourth, we focus only on AEs in one reaction group, and the deep relationship between semaglutide and other system organ classes remains unknown. Further experimental exploration, clinical trials, case-control studies, and cohort studies are needed to validate the results.

## Conclusion

Our pharmacovigilance study provides the most updated analysis between semaglutide and gastrointestinal AEs based on real-world large-sample safety data. Among the 45 gastrointestinal AEs, 17 new and unexpected AEs signals are detected. Patient age (*p* < 0.001) and body weight (*p* = 0.006) rather than sex (*p* = 0.251) might be associated with an increased risk of gastrointestinal AEs severity. One strong, 22 moderate and 22 weak clinical priority signals were defined. The median TTO for strong, moderate and weak clinical priority signals were 23, 6, and 7 days, respectively. All of the disproportionality signals had early failure type features, suggesting that the majority of patients developed gastrointestinal AEs within 1 week or 1 month of semaglutide treatment, and that the risk of gastrointestinal AEs occurrence gradually decreased over time. Our results would potentially prompt improved awareness of semaglutide-related toxicities and provide valuable references for healthcare professionals to mitigate the risk of gastrointestinal AEs by post-marketing safety assessments.

## Data availability statement

The original contributions presented in the study are included in the article/Supplementary material, further inquiries can be directed to the corresponding author.

## Author contributions

QZ and YS contributed to conception and study design, and took responsibility for the collection, integrity, and accuracy of the data. All authors drafted the manuscript, participated in data analyses and interpretation, revisions of the manuscript, and approved the final version.

## Funding

This study was supported by grants from National Natural Science Foundation of China (No. 82104476).

## Conflict of interest

The authors declare that the research was conducted in the absence of any commercial or financial relationships that could be construed as a potential conflict of interest.

## Publisher's note

All claims expressed in this article are solely those of the authors and do not necessarily represent those of their affiliated organizations, or those of the publisher, the editors and the reviewers. Any product that may be evaluated in this article, or claim that may be made by its manufacturer, is not guaranteed or endorsed by the publisher.
