# Supplementary material for: Gastrointestinal adverse events associated with semaglutide: A pharmacovigilance study based on FDA adverse event reporting system
Source: Front Public Health. 2022 Oct 20;10:996179. doi: 10.3389/fpubh.2022.996179 (PMC9631444; doi:10.3389/fpubh.2022.996179)
Supplement: Supplementary file 1 [file Table_1.DOCX]

**Supplementary Table S1**. Calculation of reporting odds ratio (ROR).

|  | Reports with target AE | Reports without target AE |
| --- | --- | --- |
| Reports with semaglutide | a | b |
| Reports without semaglutide | c | d |

a, number of reports containing both the target drug (semaglutide) and target AE; b, number of reports containing other AEs of the target drug; c, number of reports containing the target AE of other drugs; d, number of reports containing other drugs and other AEs.

AEs, Adverse Events; ROR, Reporting Odds Ratio; CI, confidence interval.

The calculation formulas are shown below:

1. ROR=ad/b/c
2. 95%CI=e^ln(ROR)±1.96(1/a+1/b+1/c+1/d)^0.5^

**Supplementary Table S2**. A rating scale assessing clinical priority of disproportionality signals.

| **Assessment items** | **2 points** | **1 point** | **0 point** |
| --- | --- | --- | --- |
| Number of target events | >50 | 10-50 | <10 |
| ROR_025_ | >5 | 2-5 | 1-2 |
| Mortality proportion | >50% | 25-50% | <25% |
| IMEs or DMEs | DME | IME | None |
| Relevant evidence evaluation | ++ | + | **-** |

Mortality proportion: percentage of cases in which death was reported as an outcome in the overall cases report for a particular adverse event. IMEs and DMEs are developed and updated by EMA (European Medicines Agency, 2020). ++ : AEs are mainly from the FDA Prescribing Information, the Summary of Product Characteristics of esketamine posted by the MHRA, Phase 2/3 RCTs, or systematic reviews, with biological plausibility. + : AEs are mainly from other clinical trials, observational studies, or case reports/series with potential biological plausibility. - : AEs only emerging from disproportionality analyses.

AEs, Adverse Events; DMEs, Designated Medical Events; IMEs, Important Medical Events; MHRA, Medicine and Healthcare Products Regulatory Agency; RCTs, Randomized Controlled Trials; ROR_025_, the lower limit of 95% confidence interval of ROR.

**Supplementary Table S3**. Top 10 combination drugs for semaglutide-associated gastrointestinal adverse events from FAERS databases.

| Combination drugs (TOP 10) | N (%) |
| --- | --- |
| METFORMIN HYDROCHLORIDE | 564 (10.36) |
| INSULIN GLARGINE | 170 (3.12) |
| ASPIRIN | 117 (2.15) |
| EMPAGLIFLOZIN | 104 (1.91) |
| ATORVASTATIN | 103 (1.89) |
| INSULIN DEGLUDEC | 95 (1.75) |
| INSULIN ASPART | 81 (1.49) |
| LISINOPRIL | 79 (1.45) |
| AMLODIPINE BESYLATE | 72 (1.32) |
| GLIPIZIDE | 72 (1.32) |

N, number of adverse event reports.

**Supplementary Table S4**. Signal strength of reports of semaglutide at the System Organ Class (SOC) level in FAERS database.

| System Organ Class (SOC) | Semaglutide cases  reporting SOC | ROR(95% CI) |
| --- | --- | --- |
| Gastrointestinal disorders* | 5,442 | 4.21 (4.06-4.37) |
| General disorders and administration site conditions | 3,670 | 0.76 (0.73-0.79) |
| Injury, poisoning and procedural complications | 3,088 | 0.89 (0.85-0.93) |
| Nervous system disorders | 2,396 | 1.05 (1.00-1.10) |
| Investigations* | 2,265 | 1.93 (1.85-2.02) |
| Metabolism and nutrition disorders* | 1,924 | 2.73 (2.60-2.87) |
| Vascular disorders | 1,378 | 0.80 (0.75-0.84) |
| Skin and subcutaneous tissue disorders | 1,322 | 0.68 (0.64-0.72) |
| Psychiatric disorders | 1,107 | 0.71 (0.67-0.75) |
| Cardiac disorders | 1,001 | 0.79 (0.74-0.84) |
| Respiratory, thoracic and mediastinal disorders | 856 | 0.48 (0.45-0.52) |
| Musculoskeletal and connective tissue disorders | 823 | 0.58 (0.54-0.62) |
| Eye disorders* | 765 | 1.64 (1.53-1.77) |
| Infections and infestations | 626 | 0.46 (0.42-0.50) |
| Renal and urinary disorders | 557 | 0.66 (0.60-0.71) |
| Product issues | 493 | 1.01 (0.92-1.10) |
| Endocrine disorders* | 481 | 1.75 (1.60-1.92) |
| Immune system disorders | 419 | 0.35 (0.32-0.39) |
| Hepatobiliary disorders | 348 | 1.11 (1.00-1.24) |
| Surgical and medical procedures | 268 | 0.70 (0.62-0.79) |
| Neoplasms benign, malignant and unspecified | 262 | 0.23 (0.20-0.26) |
| Reproductive system and breast disorders | 178 | 0.32 (0.28-0.38) |
| Ear and labyrinth disorders | 137 | 0.97 (0.82-1.15) |
| Social circumstances | 83 | 0.58 (0.47-0.72) |
| Blood and lymphatic system disorders | 80 | 0.13 (0.11-0.16) |
| Pregnancy, puerperium and perinatal conditions | 33 | 0.15 (0.11-0.22) |
| Congenital, familial and genetic disorders | 5 | 0.07 (0.03-0.18) |

* indicates statistically significant signals in algorithm. ROR, reporting odds ratio; CI, confidence interval.
